# Global Trade Pattern of Medical Devices and China's Trade Position: Based on Data From 2001 to 2020

**DOI:** 10.3389/fpubh.2022.926216

**Published:** 2022-07-22

**Authors:** Hao Hu, Xiaoping Wang, Haiyan Zhou, Zhuang Jin, Shaobin Wei

**Affiliations:** ^1^School of Economics, Shanghai University, Shanghai, China; ^2^Business School, NingboTech University, Ningbo, China; ^3^Institute of Artificial Intelligence and Change Management, Shanghai University of International Business and Economics, Shanghai, China; ^4^Baotou Teachers' College, Inner Mongolia University of Science and Technology, Baotou, China; ^5^Institute of Spatial Planning and Design, Zhejiang University City College, Hangzhou, China

**Keywords:** medical device trade, complex network, topological structure, spatiotemporal pattern, interdependence

## Abstract

To depict the evolution of the global trade of medical devices, this study analyzes the spatiotemporal evolution characteristics of global and China's trade patterns of medical devices from 2001 to 2020 based on data from the World Bank and United Nations Commodity Trade Statistics Database, and thereby investigates the status quo of global and China's medical device trade, as well as changes in China's position in the global medical device trade. The findings are as follows. First, the total global trade volume of medical devices is generally on the rise, showing closer network connections. Despite some changes in trade position, the core countries in the global medical device trade network are relatively fixed. The intermediate position of core trading countries has been weakened on the whole, whereas exporting countries have generally assumed an enhanced central position. Communities with geographical proximity have been formed in the global medical device trade network, including two large communities, the Asian-European countries and the Pacific Rim countries, and one small community, the South American countries. Second, with its rapidly growing trade volume of medical devices with other countries, China has now become the fourth largest medical device trading country in the world. Its number of import and export partners has remained relatively stable and continued to increase. Its export markets are relatively concentrated, and a tripartite pattern of import sources has been formed. China has established extensive interdependent relations and almost no one-way dependent relations in the medical device trade. Among its major trading partners for medical devices, the interdependence of China with developed countries/regions, such as European and American countries and Japan, has generally deepened.

## Introduction

Since the reform and opening up of China, Chinese people's awareness of life and health has increased with the improvement of living standards, and the state has paid more and more attention to the healthcare industry. As an important part of the healthcare system reform, the medical device industry is facing important challenges. Medical devices are an important component of human health protection, and the most important basic element in the construction of the medical service system. It is also a knowledge-intensive, highly specialized, and interdisciplinary high-tech industry. In recent years, with the continuous introduction of new medical reform policies in China, the healthcare system reform has attracted widespread attention.

In 2020, the COVID-19 pandemic had a severe impact on international trade and the world economy, posing serious challenges to the global public health system. As the basic equipment for modern healthcare, medical devices have played an important role in the global fight against the pandemic. Moreover, they are also products related to livelihood and health. As the foundation of the modern healthcare system, the development of medical devices is related to the future development of the national health industry. During the COVID-19 pandemic, medical devices, as one of the most important sectors of the healthcare industry, are an important measure of a country's scientific and technological progress, which further highlights its importance to a country's healthcare system. It is foreseeable that countries around the world will pay more and more attention to the medical device industry. In the long run, the outbreak of this pandemic is beneficial to the development of the medical device industry.

## Theoretical Basis

The international trade network is a complex economic system composed of interconnected national or regional economies. It is a new hot topic in the field of international trade. It has been studied from different disciplines and perspectives. Most studies investigated the topological structure and characteristics of the trade network based on centrality, community, clustering coefficient, and other measures of the whole industry or a certain sector or product around the world or in a certain region in a specific year or a long period of time through complex network analysis using a binary or weighted network (1–9).

Research on medical devices mainly takes a micro perspective and examines the technological innovation and enterprise efficiency of medical device companies using tools such as case analysis, data envelopment analysis (DEA), and questionnaire survey (10–13). Some studies have also been conducted from the perspective of the industry by investigating the current regulation status, innovation, regulation mechanisms, technical standards, and industry development of medical devices (14–20). However, few studies have been conducted from the perspective of trade.

It is evident from literature review that trade network has become the forefront of theoretical research of social network. Previous studies investigated the characteristics and patterns of trade networks by constructing a binary matrix or weighted directed network, which provides valuable insights for this study. However, further research is still required in this field. First, few studies have looked at the pattern of medical device trade network from the perspective of overall network and research findings on changes in China's position are inadequate. Second, data mining that covers the entire time scale and reflects the evolution process of global medical device trade network needs to be further expanded. The marginal contributions of this study are as follows. This study breaks the linear logic and considers both time and space dimensions. It attempts to characterize the evolution of the global medical device trade network by constructing a 20-year evolution diagram of this network and analyze the pattern of changes in China's position in medical device trade in an objective and comprehensive manner based on global medical device trade data from 2001 to 2020. It is hoped that the results of this study will provide a theoretical basis and decision-making support for China's efforts to cope with the changes in medical device trade pattern and build a medical device trade network system.

## Methodology And Data

### Methodology

#### First, Descriptive Analysis of Trade

The world and China's medical device trade trends are analyzed based on changes in total trade volume.

#### Second, Social Network Analysis

Social Network analysis is employed to examine changes in the measures of the global trade network of medical devices, including network density, average shortest path length, clustering coefficient, centrality, in-degree and out-degree, closeness centrality, betweenness centrality, and trade network group, thereby revealing the evolution characteristics of this network.

#### Third, Interdependence Index Analysis

To describe the interdependence of China with other countries in the global medical device trade, this study proposes an interdependence equation for medical device trade by drawing on the Grubel-Lloyd index that estimates the intensity of intra-industry trade:


(1) $$D\text{r}GL_{i,j}=\left[1-\left(\frac{\left|DE_{i\to j}-DI_{i\to j}\right|}{DE_{i\to j}+DI_{i\to j}}\right)\right]$$

where *DE*_*i*→*j*_ is the export of medical devices from country *i* to country *j*; *DI*_*i*→*j*_ is the import of medical devices of country *i* from country *j*; and *D*r*GL*_*i, j*_ is the interdependence index between country *i* and country *j* in medical device trade, with a value range of [0–1]. If country *i* only exports/imports medical devices to/from country *j*, there is only a one-way dependence index between the two countries, and *D*r*GL*_*i, j*_ is 0. If country *i*'s exports to country *j* are equal to its imports from country *j*, then the two countries have the greatest trade overlap, and *D*r*GL*_*i, j*_ is 1. The larger the *D*r*GL*_*i, j*_, the higher the interdependence index between the two countries in medical device trade. *D*r*GL*_*i, j*_≥0.5 indicates high interdependence between the two countries in medical device trade, 0.2<*D*r*GL*_*i, j*_ <0.5 indicates moderate interdependence, and *D*r*GL*_*i, j*_≤0.2 indicates low interdependence.

### Data

Based on existing research (21, 22), this study analyzes the global trade of medical devices in detail through empirical study of import and export data of common categories of medical devices defined under Chinese Harmonized System (HS) codes 9018, 9019, 9020, 9021, 9022, and 9402.

With countries/regions involved in the trade of medical devices abstracted as nodes, 80 countries/regions, such as mainland China, the United States, Germany, and South Korea, are selected as the research objects. Given that the imports and exports of medical devices of these countries/regions in 2001–2020 accounted for 99.05% of the world's total, relevant data are highly representative. An 80^*^80 matrix was created for the trade network of medical devices based on the bilateral trade flows of the 80 countries/regions. The characteristics of this network were analyzed with UCINET software and visualization was performed using Gephi.

## Result Analysis

### Structural Characteristics and Evolution of Global Medical Device Trade

From 2001 to 2020, the global trade volume of medical devices increased rapidly from US$112.963 billion to US$488.256 billion, representing an average annual growth of 7.23%. Changes in total trade volume are the combined result of changes in participating economies and trade volumes. With the continuous expansion of the global trade of medical devices, the number of participants in the trade has been increasing, and the structure of the trade network has become increasingly complex. From 2001 to 2020, the number of participants in the global medical device trade increased from 190 to 230, and the number of trade connections increased from 5,434 to 5,640, representing an increase of 17.39.0% and 6.65%, respectively. The global medical device trade network shows increasing complexity and has spread to all corners of the world.

#### Numerous Countries Participating in the Global Medical Device Trade and Forming a Closely Connected Network

As observed from the temporal evolution of node centrality and network connectivity (Table 1), the global medical device trade network is characterized by asymmetric structures of out-degree and in-degree of nodes and overall increasingly close connection. However, it also has the characteristics of a small-world network: a high clustering coefficient and a small average characteristic path length. Details are given as follows.

**Table 1:** Measures of global medical device trade network.

**Category**	**Measure**	**2001**	**2003**	**2005**	**2008**	**2010**	**2013**	**2015**	**2018**	**2020**
Node centrality	Average node degree	36.28	39.40	40.72	45.78	45.25	47.53	46.25	47.55	46.95
	Out-degree centralization	67.20%	64.00%	62.54%	58.66%	59.37%	56.82%	57.72%	56.08%	56.42%
	In-degree centralization	35.16%	37.08%	36.90%	35.59%	37.57%	40.15%	42.33%	36.85%	37.19%
Network connectivity	Network density	0.3769	0.4093	0.4274	0.472	0.4707	0.4924	0.4858	0.5084	0.5158
	Weighted clustering coefficient	0.499	0.519	0.533	0.560	0.557	0.574	0.569	0.584	0.586
	Average characteristic path length	1.539	1.519	1.501	1.471	1.465	1.453	1.455	1.431	1.419

First, the number of countries participating in the global medical device trade has increased, but importer and exporter countries have obviously asymmetric structures. From 2001 to 2020, the average number of trading partners of each country increased from 36 to 47, reflecting the trend of increasing trading partners of participating countries. However, the out-degree and in-degree centralization of the medical device trade network are asymmetric, but the gap narrowed slightly. Out-degree centralization decreased from 0.67 to 0.56, while in-degree centralization scores were mostly <0.4.

Second, countries are relatively closely connected in the global medical device trade, representing an integrated trade pattern. In terms of network connectivity, network density in 2020 is 0.5158, which is significantly higher than the 0.3769 in 2001. This means that the trading partners of each country are relatively concentrated, and countries have become more closely connected in medical device trade and formed a dense network, representing an integrated pattern of global medical device trade.

Third, the local clustering of the global medical device trade network has been increasing, and the trade efficiency has improved. From 2001 to 2020, the average clustering coefficient of the global medical device trade network showed an overall increasing trend and remained above 0.5, indicating that more and more trading countries have overlapping “circles of friends.” Meanwhile, the average characteristic path length gradually decreased and approached 1.4. This means that only one intermediate country is needed to achieve network connectivity between the trading countries, reflecting the high efficiency of trade realization in the global medical device trade network.

#### Relatively Fixed Core Countries in the Global Medical Device Trade Network, With a Shift From Intermediate to Central Position

##### Relatively Fixed Core Trading Countries, With Some Changes in Trade Position

Table 2 shows the ranking of trading countries/regions based on the imports and exports of medical devices in some years. It can be seen that the core exporters and importers in the global medical device trade network are relatively fixed, but there are some changes in trade position. In terms of out-strength, the core exporters were the United States, Germany, France, Switzerland, Ireland and China. Among them, the trade positions of France and Japan declined, that of China improved, and those of the United States and Germany were relatively stable. In terms of in-strength, the United States, Germany, Japan, the Netherlands, France, and China were the largest importers of medical devices in the world. Among them, the trade positions of the United States, Germany, and the Netherlands were relatively stable, while that of China remarkably improved.

**Table 2:** Changes in the top ten ranking of economies based on node strength in the global medical device trade network.

**Rank**	**2001**		**2005**		**2010**		**2015**		**2020**	
	**Out-strength**	**In-strength**	**Out-strength**	**In-strength**	**Out-strength**	**In-strength**	**Out-strength**	**In-strength**	**Out-strength**	**In-strength**
1	United States of America	United States of America	United States of America	United States of America	United States of America	United States of America	United States of America	United States of America	United States of America	United States of America
2	Germany	Germany	Germany	Netherlands	Germany	Germany	Germany	Germany	Germany	Germany
3	Japan	Japan	Netherlands	Germany	Netherlands	Netherlands	Netherlands	Netherlands	Netherlands	Netherlands
4	Netherlands	Netherlands	Ireland	Japan	France	Japan	China	China	China	China
5	France	France	France	France	Switzerland	France	Belgium	Japan	Ireland	Japan
6	Ireland	United Kingdom	Switzerland	United Kingdom	Ireland	China	Ireland	Belgium	Mexico	France
7	United Kingdom	Italy	United Kingdom	Italy	Belgium	Belgium	Switzerland	France	Switzerland	Belgium
8	Switzerland	Canada	Japan	Belgium	China	United Kingdom	Mexico	United Kingdom	Belgium	United Kingdom
9	Mexico	Belgium	Mexico	Canada	Japan	Italy	France	Italy	Singapore	Italy
10	Belgium	China	Belgium	China	Mexico	Canada	Japan	Canada	France	Canada

##### A Weakened Intermediate Position of Core Trading Countries and an Enhanced Central Position of Exporting Countries

As shown by the spatiotemporal variations in node betweenness centrality and closeness centrality (Table 3), the betweenness centrality of the core trading countries of medical devices decreased, but the closeness centrality of exporting countries increased significantly. This means that despite the weakening of exporters' control over the trade network, core exporters still maintained a strong influence due to their enhanced central position. Furthermore, horizontal comparison shows that the betweenness centrality of each country was generally low and most of the top 10 countries were core exporters, including the United States, European countries, such as France and Germany, and Asian countries, such as China and South Korea. Among them, the United States has long been ranked first in terms of betweenness centrality, reflecting that it plays a significant bridging role in the global medical device trade and has absolute control over the global trade network. It should be noted that under the integrated pattern of the global medical device trade network, the United States' trade control decreased from 8.469 in 2001 to 6.381 in 2020. On the other hand, contrary to the decrease of betweenness centrality, most of the core exporters showed an increased closeness centrality. Among them, China's export influence increased significantly, with its ranking improving from outside the top ten to the fourth in terms of closeness centrality. On the whole, the weakened intermediate position of exporters reduced their trade control, whereas their enhanced central position intensified competition among them. This may offer opportunities for medical device importers, such as China, to enhance their control over the global medical device trade.

**Table 3:** Changes in the top 10 ranking of economies based on centrality in the global medical device trade network.

**Measure**	**Year**	**1**	**2**	**3**	**4**	**5**	**6**	**7**	**8**	**9**	**10**
Betweenness centrality	2001	United States of America	Germany	Italy	France	Netherlands	Japan	Belgium	United Kingdom	Spain	Russian Federation
		8.469	7.08	4.622	4.017	3.837	3.034	2.724	2.711	1.675	1.588
	2005	United States of America	Germany	France	Italy	Netherlands	Belgium	Switzerland	United Kingdom	Russian Federation	Brazil
		9.8	6.062	4.141	3.042	3.012	2.877	2.677	1.843	1.765	1.623
	2010	United States of America	Germany	France	Italy	Switzerland	United Kingdom	Netherlands	Belgium	Egypt	China
		10.721	6.929	3.865	3.341	2.872	2.747	2.637	2.196	1.416	1.341
	2015	United States of America	Germany	France	Netherlands	Switzerland	Belgium	United Kingdom	Italy	Russian Federation	Turkey
		7.911	7.059	4.312	3.148	3.067	2.651	2.55	1.906	1.589	1.388
	2020	Germany	United States of America	Netherlands	China	France	Italy	United Kingdom	Switzerland	Belgium	Brazil
		6.786	6.381	4.659	2.724	2.612	2.525	1.755	1.752	1.603	1.569
In-degree closeness centrality	2001	United Arab Emirates	Iran, Islamic Republic of	Pakistan	Bangladesh	Kuwait	Lebanon	Panama	Ethiopia	Nigeria	Ecuador
		7.411	7.369	7.363	7.342	7.328	7.328	7.308	7.295	7.295	7.268
	2005	Kuwait	Iran, Islamic Republic of	Egypt	Bangladesh	Algeria	Panama	Nigeria	Ethiopia	Qatar	United States of America
		10.408	9.553	9.518	9.416	9.382	9.36	9.36	9.349	9.294	8.977
	2010	United Arab Emirates	Kazakhstan	Kuwait	Algeria	Germany	United States of America	France	United Kingdom	Italy	Netherlands
		22.191	21.644	21.237	21.237	19.363	19.315	18.854	18.72	18.72	18.676
	2015	Ecuador	Algeria	Venezuela, Bolivarian Republic of	Ethiopia	Germany	United States of America	France	Netherlands	Belgium	Turkey
		21.884	21.644	21.067	20.735	19.603	19.363	19.175	19.036	18.9	18.9
	2020	Algeria	Iran, Islamic Republic of	Bangladesh	Lebanon	Ethiopia	Venezuela, Bolivarian Republic of	Germany	United States of America	Netherlands	France
		15.076	15.076	15.019	14.934	14.739	14.549	14.057	13.958	13.86	13.835
Out-degree closeness centrality	2001	Germany	United States of America	France	Netherlands	Italy	United Kingdom	Belgium	Switzerland	Japan	Sweden
		50	50	49.686	49.686	49.375	49.068	48.466	48.466	48.171	47.024
	2005	United States of America	Germany	Netherlands	United Kingdom	France	Italy	Switzerland	China	Belgium	Japan
		98.75	98.75	98.75	98.75	97.531	97.531	97.531	97.531	91.86	91.86
	2010	Germany	United States of America	Netherlands	Belgium	Switzerland	France	United Kingdom	Italy	China	Korea, Republic of
		100	100	100	98.75	98.75	97.531	97.531	97.531	97.531	96.341
	2015	Germany	United States of America	Netherlands	Italy	China	France	Belgium	Korea, Republic of	United Kingdom	Switzerland
		100	100	100	98.75	98.75	97.531	97.531	97.531	96.341	96.341
	2020	Germany	United States of America	Netherlands	China	Italy	United Kingdom	Switzerland	France	Belgium	Korea, Republic of
		100	100	100	100	97.531	97.531	97.531	96.341	96.341	96.341

#### Formation of Communities With Geographical Proximity in the Global Medical Device Trade Network

**Figure 1:**
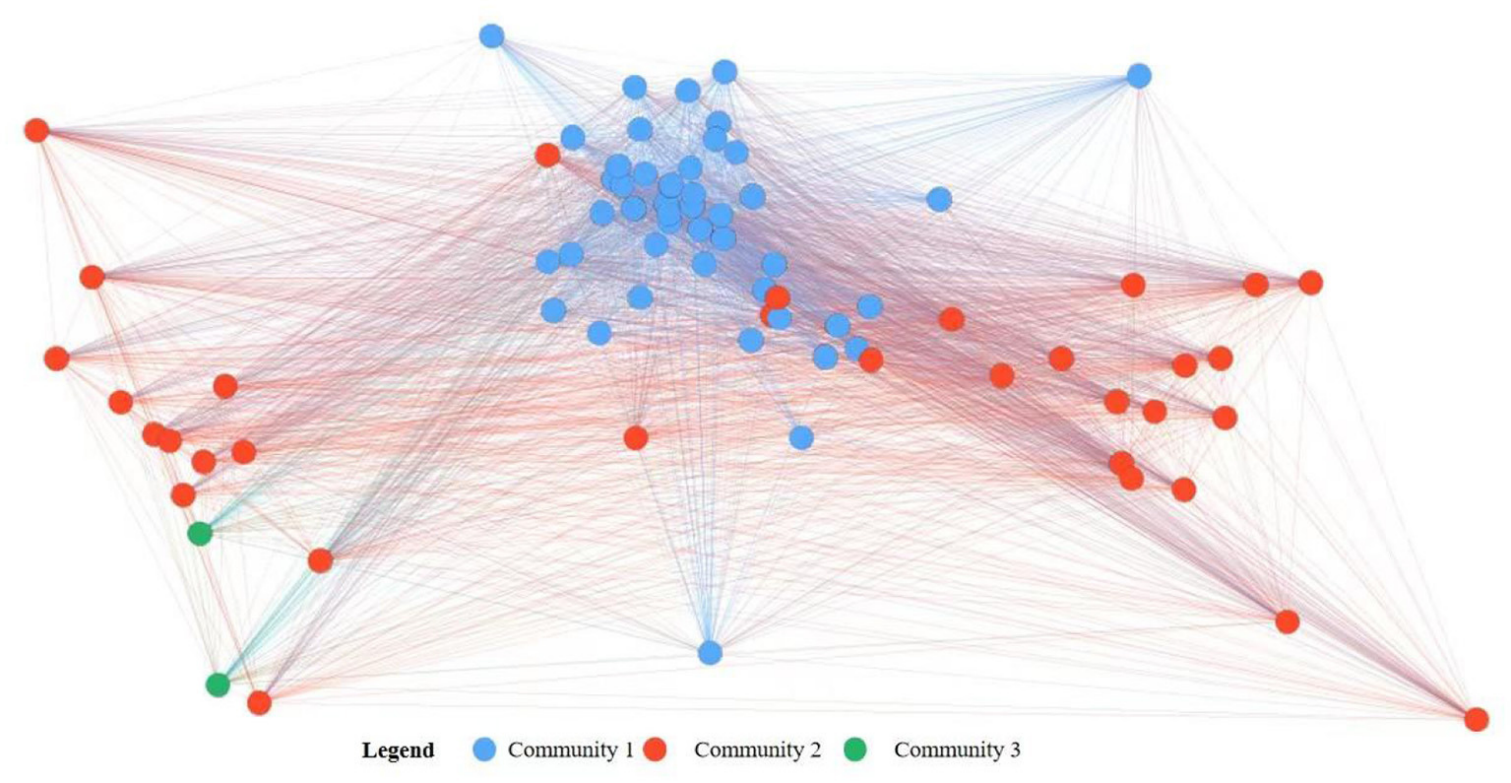
Community detection in the global medical device trade network in 2020.

The network structure and core node characteristics of the global medical device trade show the pattern of a polycentric seller's market. To further clarify the community distribution characteristics in the trade of medical devices, this study identified the communities in the directed network of global medical device trade in 2020 as an example. Using the Louvain community detection algorithm, three communities reflecting significant geographical proximity were identified: the Asian-European countries (community 1), the Pacific Rim countries (community 2), and the South American countries (community 3; Figure 1).

Community 1 is dominated by Asian and European countries. It is the community with the largest trade volume and the largest number of trade relations. It is also a main export area of medical devices. In particular, the exports of Germany and the Netherlands both exceeded US$ 4 billion. The medical device exports of its member countries accounted for 46.53% of the world's total. The trade relations established with them accounted for 58.12% of the total relations in the global medical device trade network. And the trade relations within the community accounted for 57.61%. In addition, this community includes many major importers of medical devices in the world. Countries with imports of more than US$ 100 million, such as the United Kingdom, Italy, Slovakia, and Romania, accounted for 40% of the total importers within the community. Community 2 is centered around the United States and China, covering 33 countries/regions in Asia, Europe and America. The medical device exports of its member countries accounted for 53.13% of the world's total, and their export relations accounted for 40.43%. The United States and China are the first and second core nodes, respectively, and Japan, a major exporter, is the third core node. Community 3 only includes Chile and Peru and accounted for the smallest proportion of imports (0.26%) and exports (0.61%).

### Characteristics of China's Medical Device Trade in the Network

China has been a net exporter of medical devices since 2001. Its trade surplus gradually increased from US$153 million in 2001 to US$25.747 billion in 2020. In particular, its trade surplus in 2020 increased by US$10.2 billion compared with 2018 due to the COVID-19 pandemic. China has now become the fourth largest trader of medical devices in the world. It shows the following characteristics in the import and export network of medical devices.

#### Increased Imports and Exports

China experienced high growth of medical device imports and exports from 2001 to 2020 according to the node strength and ranking shown in Table 4. Its exports increased from US$1.582 billion to 41.880 billion, with its ranking improving from 16th to 4th. Its imports increased from US$1.429 billion to 16.133 billion, with its ranking improving from 16th to 4th. In 2020, due to the COVID-19 pandemic, China demonstrated its strength in the supply and demand of medical devices for the first time, resulting in a substantial increase in exports. China has a high level of participation in the global medical device trade network, with high rankings in terms of both out- and in-strength. However, obvious differences are observed between its import and export trends.

**Table 4:** China's node degrees, imports and exports, and rankings in the medical device trade.

**Category**	**Measure**	**2001**	**2003**	**2005**	**2008**	**2010**	**2013**	**2015**	**2018**	**2020**
Imports and exports	Exports	15.82	25.68	49.91	110.10	134.03	202.87	235.18	318.64	418.80
	Rank by exports	16	14	11	10	8	6	4	4	4
	Imports	14.29	21.72	28.38	45.52	64.20	103.70	114.68	163.14	161.33
	Rank by imports	10	8	10	9	6	5	4	4	4
Node degree	Out-degree	64	71	77	78	77	78	78	78	79
	Rank by out-degree	12	9	7	4	7	5	4	4	2
	In-degree	33	34	35	41	40	42	45	50	51
	Rank by in-degree	15	22	22	14	16	16	11	7	7

#### Relatively Concentrated Export Markets and a Tripartite Pattern of Import Sources

Table 5 shows the inter-annual changes in China's major export markets for medical devices and their shares. It can be seen that the share of its top 10 export destinations in its total medical device exports is decreasing year by year. Although the United States, Japan, and Germany remain the main export markets for Chinese medical devices, their market share is decreasing year by year, representing a sharp decrease from 63.91% in 2001 to 34.59% in 2020 of China's total medical device exports.

**Table 5:** Changes in China's major export markets for medical devices and their shares.

**Rank**	**2001**	**Share**	**2005**	**Share**	**2010**	**Share**	**2015**	**Share**	**2020**	**Share**
1	United States of America	30.12%	United States of America	22.49%	United States of America	23.74%	United States of America	24.26%	United States of America	22.65%
2	Japan	28.90%	Japan	18.32%	Japan	11.65%	Japan	8.83%	Germany	6.34%
3	Germany	4.89%	Germany	6.25%	Germany	6.83%	Hong Kong, China	6.40%	Hong Kong, China	5.91%
4	Hong Kong, China	4.46%	Singapore	4.61%	Hong Kong, China	4.44%	Germany	6.11%	Japan	5.59%
5	United Kingdom	4.18%	Hong Kong, China	4.18%	Netherlands	3.39%	South Korea	3.11%	United Kingdom	3.82%
6	Netherlands	3.14%	Netherlands	3.22%	United Kingdom	2.86%	Netherlands	3.00%	South Korea	3.78%
7	Singapore	3.11%	South Korea	2.51%	India	2.28%	United Kingdom	2.96%	Hungary	2.91%
8	France	1.57%	United Kingdom	1.95%	Russia	2.25%	India	2.82%	Netherlands	2.87%
9	Italy	1.44%	Russia	1.92%	France	2.23%	Singapore	2.53%	Russia	2.56%
10	India	1.28%	Ireland	1.64%	Singapore	2.18%	Australia	1.95%	Brazil	2.42%
Total		83.09%		67.08%		61.85%		61.98%		58.86%

On the other hand, changes in the in-degree value and ranking of China's medical device trade followed a basically similar trend with those in out-degree. From 2001 to 2020, the number of import sources of China increased from 33 to 51, with the in-degree ranking rising from 15th to 7th. On the whole, China has an increasing dependence on medical device imports and has an increasing number of import sources. Furthermore, based on the inter-annual changes in China's major medical device import markets and their shares in Table 6, it can be seen that China's major medical device import sources have gradually changed from North America and Europe to North America, South America, and Europe. In 2001, China had only a few core import sources of medical devices, mainly including North American, European, and East Asian countries/regions, such as Hong Kong (China), the United States, Japan, and Germany. In 2010, China's major import sources of medical devices remained basically unchanged. Meanwhile, the market share of medical devices imported from European countries, such as Germany, the United Kingdom, the Netherlands, and Switzerland, increased. These countries gradually became important import sources of medical devices for China. In 2020, Asian countries, such as Singapore (3.40%) and South Korea (2.70%), have become important import sources of medical devices for China.

**Table 6:** Changes in China's major import markets for medical devices and their shares.

**Rank**	**2001**	**Share**	**2005**	**Share**	**2010**	**Share**	**2015**	**Share**	**2020**	**Share**
1	Hong Kong, China	26.05%	United States of America	22.65%	United States of America	25.92%	United States of America	28.62%	United States of America	28.36%
2	United States of America	25.73%	Hong Kong, China	17.22%	Germany	16.85%	Germany	15.58%	Germany	17.50%
3	Japan	18.44%	Germany	16.13%	Hong Kong, China	14.96%	Japan	8.71%	Japan	9.35%
4	Germany	10.95%	Japan	15.49%	Japan	12.68%	Singapore	8.56%	Netherlands	7.49%
5	France	2.57%	Singapore	6.48%	Netherlands	3.68%	Hong Kong, China	6.20%	Hong Kong, China	4.98%
6	Singapore	2.02%	Netherlands	3.53%	Singapore	3.02%	Netherlands	5.14%	Singapore	3.40%
7	Netherlands	1.92%	South Korea	2.56%	Switzerland	2.71%	Switzerland	3.69%	Switzerland	3.40%
8	South Korea	1.65%	United Kingdom	2.37%	United Kingdom	2.43%	Belgium	2.77%	Belgium	2.90%
9	United Kingdom	1.60%	France	2.07%	France	1.95%	South Korea	2.58%	South Korea	2.70%
10	Italy	1.17%	Switzerland	1.83%	Israel	1.80%	Israel	2.33%	Israel	2.40%
Total		92.10%		90.32%		86.00%		84.19%		82.48%

In general, China's medical device import sources have developed into a tripartite pattern consisting of North America, Europe, and Asia. Within this pattern, the main import sources include community 1 and 3 members, such as the United States, Germany, Japan, the Netherlands, Hong Kong (China), and Singapore, together maintaining a share of around 80% in China's medical device market.

### Interdependence of China in Medical Device Trade

To characterize the interdependence between China and its trading partners for medical devices, and considering the high concentration of China's medical device trade volume, this study uses the independence index model to calculate China's interdependence index with its major trading partners of medical devices in 2001, 2010, and 2020, respectively (Table 7).

**Table 7:** Interdependence between China and its top 20 trading partners for medical devices in 2001, 2010, and 2020.

**Rank**	**2001**		**2010**		**2020**	
	**Country/region**	**DrGL***_i, j_*****	**Country/region**	**DrGL***_i, j_*****	**Country/region**	**DrGL***_i, j_*****
1	United States of America	0.7861	United States of America	0.9772	United States of America	0.9818
2	Japan	0.9287	Japan	0.9791	Germany	0.6400
3	Hong Kong, China	0.1731	Germany	0.5947	Japan	0.8744
4	Germany	0.3963	Hong Kong, China	0.4724	Hong Kong, China	0.7867
5	United Kingdom	0.8190	Netherlands	0.9798	Netherlands	0.6638
6	Singapore	0.9205	United Kingdom	0.8979	South Korea	0.7103
7	Netherlands	0.9495	Singapore	0.8583	United Kingdom	0.5051
8	France	0.5048	France	0.9112	Singapore	0.7814
9	South Korea	0.4473	South Korea	0.8738	Belgium	0.7159
10	Italy	0.8127	Switzerland	0.4307	France	0.8622
11	India	0.8924	Italy	0.6184	Hungary	0.0272
12	Ireland	0.9550	India	0.4027	Switzerland	0.2032
13	Spain	0.4542	Belgium	0.9812	India	0.3857
14	Denmark	0.3822	Australia	0.5224	Israel	0.7090
15	Switzerland	0.0345	Russia	0.0295	Russia	0.0225
16	Sweden	0.1598	Denmark	0.8251	Brazil	0.0338
17	Israel	0.3352	Israel	0.3940	Italy	0.5331
18	Taiwan, China	0.3349	Sweden	0.5369	Australia	0.3163
19	Australia	0.6927	Taiwan, China	0.8578	Vietnam	0.7389
20	Canada	0.8389	Brazil	0.0344	Mexico	0.5906

In terms of medical device trade, the interdependence between China and developed countries/regions, such as the United States, Hong Kong (China) and Germany, has generally increased, while that with Japan and the United Kingdom has decreased significantly. Furthermore, for most countries/regions with high interdependence with China, China's exports to them were higher than their exports to China in 2020, which indicates that these countries/regions were more dependent on China. However, China was more dependent on these countries/regions in 2001 and 2010. Meanwhile, China's exports to countries/regions with low interdependence have gradually increased and were much higher than their exports to China, resulting in low interdependence but high one-way dependence.

According to the ranking of China's major trading partners by interdependence index (Table 8), the number of countries/regions that maintain high interdependence with China on the medical device trade has gradually increased and most of them are developed countries/regions, such as European and American countries and Japan. The number of countries/regions that maintain low interdependence with China has gradually decreased, and there have been no countries/regions that are completely one-way dependent on China.

**Table 8:** Ranking of China's top 20 trading partners for medical devices by interdependence in 2001, 2010, and 2020.

**Interdependence**	**2001**	**2010**	**2020**
High	France, Australia, United States of America, Italy, United Kingdom, Canada, India, Singapore, Japan, Netherlands, Ireland	Australia, Sweden, Germany, Italy, Denmark, Taiwan, Singapore, South Korea, United Kingdom, France, United States, Japan, Netherlands, Belgium	United Kingdom, Italy, Mexico, Germany, Netherlands, Israel, Korea, Belgium, Vietnam, Singapore, Hong Kong, France, Japan, United States of America
Medium	Hong Kong (China), Taiwan (China), Israel, Denmark, Germany, South Korea, Spain	Israel, India, Switzerland, Hong Kong (China)	Switzerland, Australia, India
Low	Switzerland, Sweden, Hong Kong (China)	Russia, Brazil	Russia, Hungary, Brazil

On the whole, China has established extensive interdependent relations and almost no one-way dependent relations in the medical device trade. Among its major trading partners for medical devices, the interdependence of China with developed countries/regions, such as European and American countries and Japan, has generally deepened.

## Conclusions And Discussions

### Conclusions

This study analyzes the spatiotemporal evolution characteristics of global and China's trade patterns of medical devices from 2001 to 2020 based on data from the World Bank and United Nations Commodity Trade Statistics Database, and thereby investigates the status quo of global and China's medical device trade, as well as changes in China's position in the global medical device trade. The findings are as follows.

First, the total global trade volume of medical devices is generally on the rise. The changes in total trade volume are the combined result of changes in participating economies and trade volumes. The development trends of the number of participating economies and trade connections generally correspond to the total trade volume. Connections between countries/regions in the medical device trade have strengthened, which is reflected by increasing interactions and interdependence, and closer network connections. Despite some changes in trade position, the core countries in the network are relatively fixed. The intermediate position of core trading countries has been weakened on the whole, whereas exporting countries have generally assumed an enhanced central position. Communities with geographical proximity have been formed in the global medical device trade network, including two large communities, the Asian-European countries and the Pacific Rim countries, and one small community, the South American countries.

Second, China's trade volume of medical devices with other countries has grown rapidly, exhibiting a continuous upward trend. With its greatly increased imports and exports, it has now become the fourth largest medical device trading country in the world. Its number of import and export partners has remained relatively stable and continued to increase. Besides, its export markets are relatively concentrated, and a tripartite pattern of import sources has been formed. According to the interdependence index, the interdependence between China and developed countries/regions, such as the United States, Hong Kong (China) and Germany, has generally increased in the medical device trade, while that with Japan and the United Kingdom has decreased significantly. China has established extensive interdependent relations and almost no one-way dependent relations in the medical device trade. Among its major trading partners for medical devices, the interdependence of China with developed countries/regions, such as European and American countries and Japan, has generally deepened.

### Discussions

Due to the COVID-19 pandemic, the global demand for medical devices has surged. In the context of the persistent pandemic, the international dependence on Chinese medical devices may further increase. Therefore, the following suggestions are made.

First, the application of big data promotes the rapid development of the medical device industry. As the first entry point for collecting patient health data, medical devices have an important strategic position. In addition to providing support for services, deep mining of health big data can also lead the strategic planning and guide the direction of future research and development. Big data mining makes it possible to reduce the workload of doctors during the pandemic, improve the efficiency of diagnosis, and improve the accuracy of diagnostic tests. Big data enables the quality upgrade and structural optimization of the medical device industry, and promotes the high-end leap of the whole industry chain. It can also optimize the allocation of resource elements in the medical device industry and improve total factor productivity. Moreover, digitalization can give birth to new models, new demands, and new forms of the medical device industry, creating new momentum for industrial growth.

Second, since the outbreak of the COVID-19 pandemic, China has demonstrated its strength in supplying medical devices and occupied the global market with numerous medical device orders. China should seize the current opportunities, dedicate more efforts to innovation and research and development, and strengthen international cooperation, especially in the field of high-end medical devices. Meanwhile, efforts should also be made to enhance the international influence of Made in China brands, improve quality standards, continuously strengthen the publicity of Chinese medical device brands, and promote overseas marketing of more pharmaceutical brands.

## Data Availability Statement

The original contributions presented in the study are included in the article/supplementary material, further inquiries can be directed to the corresponding authors.

## Author Contributions

All authors listed have made a substantial, direct, and intellectual contribution to the work and approved it for publication.

## Funding

This work was supported by the Major Program of the National Social Science Foundation of China (grant number 20&ZD124), the National Natural Science Foundation of China (grant numbers 72103129, 72173014, 71973129, 72072162, and 71773115), the Philosophy and Social Science Program of Zhejiang (grant number 22NDQN290YB), the Humanity and Social Science Foundation of Ministry of Education of China (grant number 21YJA790043).

## Conflict of Interest

The authors declare that the research was conducted in the absence of any commercial or financial relationships that could be construed as a potential conflict of interest.

## Publisher's Note

All claims expressed in this article are solely those of the authors and do not necessarily represent those of their affiliated organizations, or those of the publisher, the editors and the reviewers. Any product that may be evaluated in this article, or claim that may be made by its manufacturer, is not guaranteed or endorsed by the publisher.

## References

[1] Newman M Park J. Why social networks are different from other types of networks. Phys Rev E. (2003) 68:036122. 10.1103/PhysRevE.68.036122 14524847

[2] Serrano M Boguna M. Topology of the world trade web. Phys Rev E Stat Nonlin Soft Matter Phys. (2003) 634–46. 10.1103/PhysRevE.68.015101 12935184

[3] Mahutga M. The persistence of structural inequality? A network analysis of international trade, 1965-2000. Soc Forces. (2006) 84:1863–89. 10.1353/sof.2006.0098

[4] Fagiolo G. The international-trade network: gravity equations and topological properties. J Econ Interact Coord. (2010) 5:1–25. 10.1007/s11403-010-0061-y

[5] Krapohl S Fink S. Different paths of regional integration: Trade Networks and Regional Institution-Building in Europe, Southeast Asia and Southern Africa. J Common Mark Stud. (2013) 51:472–88. 10.1111/jcms.12012

[6] Ji Q Zhang H Fan Y. Identification of global oil trade patterns: an empirical research based on complex network theory. Energy Convers Manag. (2014) 85:856–65. 10.1016/j.enconman.2013.12.072

[7] Gephart J Pace M. Structure and evolution of the global seafood trade network. Environ Res Lett. (2015) 10:125014. 10.1088/1748-9326/10/12/125014

[8] Basile R Commendatore P Benedictis L Kubin I. The impact of trade costs on the European regional trade network: an empirical and theoretical analysis. Rev Int Econ. (2018) 26. 10.1111/roie.12314

[9] Xi X Xi BX Miao CL Yu R Xie J Xiang R. Factors influencing technological innovation efficiency in the Chinese video game industry: applying the meta-frontier approach. Technol Forecast Soc Change. (2022) 178:121574. 10.1016/j.techfore.2022.121574

[10] Svistounov A Kestell C Adams K Munday K. Effects of medical device legislation on innovation within Australian manufacturing companies. Innov Manage Policy Pract. (2007) 9:343–50. 10.5172/impp.2007.9.3-4.343

[11] Chih-Hao L Show-Ling J. The impact of M&As on company innovation: evidence from the US medical device industry. Scientometrics. (2010) 84:119–31. 10.1007/s11192-009-0096-9

[12] Cristia J. Organizing Technological Innovation of Medical Devices Companies: An Empirical Study of Two Midland Venture Companies. Leicester: University of Leicester (2014).

[13] Hong CF Lin YH. Efficiency of publicly-traded medical device companies in Taiwan - an analysis using window-DEA. In: 2015 IEEE International Conference on Industrial Engineering and Engineering Management. IEEE (2015), p. 1791–4. 10.1109/IEEM.2015.7385956

[14] Sorenson C Drummond M. Improving medical device regulation: the UnitedStates and Europe in perspective. Milbank Q. (2014) 92:114–50. 10.1111/1468-0009.12043 24597558 PMC3955380

[15] Boyer P Morshed B Mussivand T. Medical device market in China. Artif Organs. (2015) 39:520–5. 10.1111/aor.12427 25735659

[16] Zhang W Liu R Chatwin C. Chinese Medical Device Market and The Investment Vector. Papers. (2016). 10.48550/arXiv.1609.05200

[17] Lee M Yoon Y Ryu GH Bok HS Yoon K Park S. Innovative distribution priorities for the medical devices industry in the fourth industrial revolution. Int Neurourol J. (2018) 22:S83–90. 10.5213/inj.1836152.076 30068070 PMC6077940

[18] A1tayyar SS. The essential principles of safety and effectiveness for medical device and the role of standards. Med Device. (2020) 13:49–55. 10.2147/MDER.S235467 32104108 PMC7026114

[19] Petra M Ladislav H Ondrej K Michael S Kamil K. New regulations on medical devices in Europe: are they an opportunity for growth? Adm Sci. (2020) 10, 16. 10.3390/admsci10010016

[20] Shelby DR Lori M. Moving beyond the tip of the iceberg to incorporate patients' perspectives and outcomes in the medical device regulatory process. Value Health. (2020) 23:298–9. 10.1016/j.jval.2020.01.008 32197724

[21] Xi X Wei S Lin K-L Zhou H Wang K Zhou H. Digital technology, knowledge level, and food safety governance: Implications for national healthcare system. Front Public Health. (2021) 9:753950. 10.3389/fpubh.2021.753950 34900901 PMC8655841

[22] Hu F Xi X Zhang Y. Influencing mechanism of reverse knowledge spillover on investment enterprises' technological progress: an empirical examination of Chinese firms. Technol Forecast Soc. (2021) 169:120797. 10.1016/j.techfore.2021.120797

